# Cephalic Tetanus from Penetrating Orbital Wound

**DOI:** 10.1155/2009/548343

**Published:** 2009-05-24

**Authors:** Eloïse Guyennet, Jean-Laurent Guyomard, Emilie Barnay, Franck Jegoux, Jean-François Charlin

**Affiliations:** ^1^Department of Ophthalmology, CHU Pontchaillou, rue Henri le Guilloux, 35000 Rennes, France; ^2^Department of Oto-Rhino-Laryngology, CHU Pontchaillou, rue Henri le Guilloux, 35000 Rennes, France

## Abstract

Tetanus is a neurologic disorder caused by tetanospasmin, a protein toxin elaborated by Clostridium tetani. Cephalic tetanus is a localized form of the disease causing trismus and dysfunction of cranial nerves. We report the case of a man who presented with facial trauma, complete ophthalmoplegia, exophthalmos, areactive mydriasis, and periorbital hematoma. An orbital CT revealed air bubbles in the right orbital apex. The patient was given a tetanus toxoid booster and antibiotherapy. After extraction of a wooden foreign body, the patient developed right facial nerve palsy, disorders of swallowing, contralateral III cranial nerve palsy, and trismus. Only one case of cephalic tetanus from penetrating orbital wound has been reported in literature 20 years ago. When a patient presents with an orbital wound with ophthalmoplegia and signs of anaerobic infection, cephalic tetanus should be ruled out.

Tetanus is a neurologic disorder caused by tetanospasmin, a powerful protein toxin produced by *Clostridium tetani*. It is a rare disease in industrialized countries because of vaccination. Cephalic tetanus is a localized form of the disease with trismus and dysfunction of one or more cranial nerves [1].

A 79-year-old, with unknown vaccine status, presented with a facial trauma after a fall in a moat. Initial examination revealed, on the right side, complete ophthalmoplegia, exophthalmos, areactive mydriasis, and periorbital hematoma. Visual acuity of the right eye was hand motion and 20/25 in the left eye. The ophthalmic examination of the right eye was normal, without penetrating wound. The initial orbital computed tomography (CT) revealed intra- and extra-conal air bubbles in the right orbital apex (Figures 1(a)  and 1(b)).

A transconjunctival surgical exploration allowed drainage of a retro-ocular purulent collection and extraction of a 5 mm long wooden foreign body. After bacteriological analysis, the patient was given a tetanus toxoid booster (immunoglobulins + vaccine) and placed on intravenous amoxicillin/clavulanate 1g every eight hours. 

By the third postoperative day, the examination revealed the persistence of ophthalmoplegia and an increase of the exophthalmos. An orbital MRI revealed an increase of the orbital abscess (Figures 2(a)  and 2(b)). A second orbitotomy allowed drainage of the abscess. Cultures revealed *Enterococcus faecalis*, *Clostridium perfringens*, *Staphylococcus epidermidis,* and *Streptococcus A*. No *Clostridium tetani* was found.

The following day, the patient had a right facial nerve palsy, disorders of swallowing, contralateral III cranial nerve palsy, and trismus. The diagnosis of cephalic tetanus was retained. The patient was hospitalized in intensive care unit where he received aggressive supportive care, penicillin and tetanus immunoglobulins. The patient recovered and was discharged from the intensive care unit 2 weeks after his admission. 

Only one case of cephalic tetanus from penetrating orbital wound has been reported in literature 20 years ago [2]. To our knowledge, it is the first case which reported cephalic tetanus unless seroprevention had been done. 

Tetanus is an infectious bacterial disease caused by *Clostridium tetani*, a gram-positive anaerobic bacillus that is ubiquitous in soil. When inoculated into oxygen-poor sites, such as necrotic tissue, *C. tetani* spores germinate to vegetative bacilli that multiply and elaborate tetanospasmin, a potent neurotoxin. The toxin migrates across the synapse to presynaptic terminals, where it blocks the release of the inhibitory neurotransmitters glycine and GABA. This involvement of the neuromuscular junction causes weakness and paralysis [1]. It is a rare disease in the developed world. 

Clinical tetanus comprises four symptomatic types: generalized, local, cephalic, and neonatal tetanus [1]. The diagnosis of tetanus is based entirely on clinical findings. *C. tetani* can be isolated from wounds of patients without tetanus and frequently cannot be recovered from wounds of those with tetanus [1].

Cephalic tetanus is a rare form of tetanus defined as trismus and paralysis of one ore more cranial nerves. The most frequently involved cranial nerve is the facial nerve. The incidence of cephalic tetanus ranges from 1% to 3% of all forms of tetanus [3]. Two thirds of cephalic tetanus cases progress to generalized tetanus. The mortality is high: 15 to 30% [3].

Treatment involves debridement of wounds, administration of penicillin and tetanus immunoglobulins, aggressive supportive care, and initiation of active immunization [4].

All partially immunized and unimmunized adults should receive vaccine. For prevention, the primary series for adults consists of three doses. A booster dose is required every ten years [5].

Some patients with wounds require, in addition to the vaccine, passive immunization with tetanus immunoglobulin. It is not recommended in cases of clean minor wounds [1]. For other wounds the recommendations are tetanus toxoid booster if the patient has completed the primary series of three doses of tetanus toxoid but the last dose dates back more than 5 years, or tetanus toxoid booster plus immunoglobulins if the patient has an unknown history of immunization or has not completed the primary series [5].

When a patient presents with an orbital wound and ophthalmoplegia, a differential diagnosis of cephalic tetanus should be ruled out. Tetanus immunization status should be ascertained in all patients with potentially contaminated wounds in and around the eye. 

## Figures and Tables

**Figure 1:**
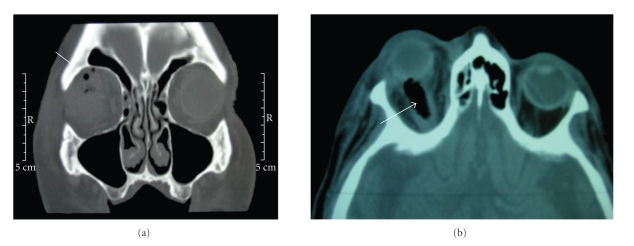
CT at the admission. (a) The arrow indicates extraconal air bubbles, (b) intra-conal air bublle.

**Figure 2:**
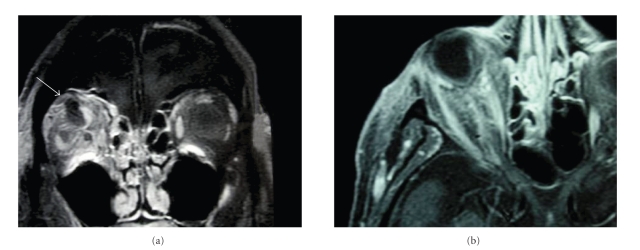
Cephalic and orbital MRI. (a) The arrow shows a hyposignal corresponding to the abscess, (b) exophthalmos stage I.
